# Assessment of lead and cadmium exposure through olive and corn oil consumption in Gonbad-Kavus, north of Iran: A public health risk analysis

**DOI:** 10.1016/j.toxrep.2025.101922

**Published:** 2025-01-21

**Authors:** Janan Tayeb, Mohammadhosein Movassaghghazani

**Affiliations:** a Faculty of Veterinary Medicine, Shabestar Branch, Islamic Azad University, Shabestar, Iran; b Department of Food Hygiene and Quality Control, Faculty of Veterinary Medicine, Shabestar Branch, Islamic Azad University, Shabestar, Iran

**Keywords:** Olive oil, Corn oil, Lead, Cadmium, Public health risk analysis, Iran

## Abstract

Lead and cadmium are common heavy metals in oils. This study assessed their levels in commercial and traditional olive and corn oils from Gonbad-Kavus City using graphite furnace atomic absorption spectrometry after microwave digestion. Hazard Quotient (HQ) and Hazard Index (HI) were calculated. The results from 60 oil samples showed quantifiable levels of lead and cadmium in all samples. Lead concentrations in commercial olive oil, traditional olive oil, commercial corn oil, and traditional corn oil were 13.27 ± 3.37, 17.48 ± 4.82, 19.27 ± 8.12, and 32.40 ± 6.13 μg/kg, respectively. Cadmium concentrations were 4.14 ± 0.53, 3.50 ± 0.72, 4.48 ± 1.80, and 5.77 ± 1.34 µg/kg, respectively. All lead levels were below the 80 µg/kg limit set by the Institute of Standards and Industrial Research of Iran (ISIRI). For a 70 kg person consuming 0.147 g of corn oil and 0.328 g of olive oil daily, the metals pose no risk to health over a lifetime. No health concerns were found for oils except traditional olive oil. Corn oil showed significant lead contamination. HI values for lead and cadmium in oils were below 1, indicating no non-carcinogenic health risk. MOE values for lead in traditional olive oil were below 10,000, while other oils were above, indicating no significant risk to consumers. These findings call for a review of national standards and increased monitoring of heavy metals in vegetable oils in the region.

## Highlights

• Lead and cadmium were detected in all tested oil samples.
• Corn oil showed significant lead contamination.
• No health risk for most oils; potential risk in traditional olive oil.
• Hazard index values below 1 indicate no non-carcinogenic risk.
• Continuous monitoring and review of standards recommended.

## Graphical Abstract

**Figure:**
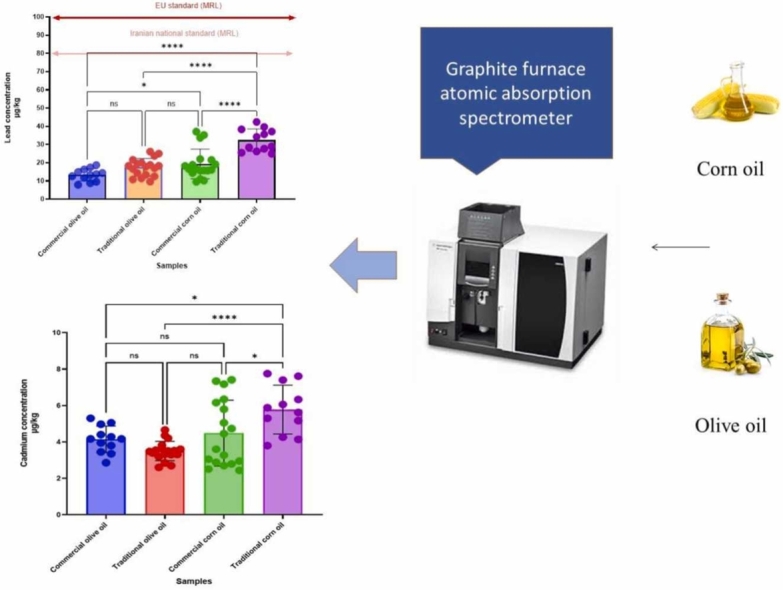

## 1 Introduction

Vegetable oil is widely used in several industries, including food processing, chemicals, pharmaceuticals, and cosmetics, globally [1]. Contamination of vegetable oils with contaminants and farming-related compounds, including metals and pesticides, is a major concern. The environment contains naturally occurring potentially hazardous elements, such as heavy metals and trace elements (1).

Heavy metals in food from industrialization pose a significant health threat [2]. Heavy metals (such as mercury, chromium, lead, arsenic, and cadmium) can enter the food chain system through a variety of means, including sewage, dust, contaminated soils, and industrial waste. Additionally, the body produces poisonous heavy metals through the substitution of necessary metal ions in chelates. According to numerous studies, lead and cadmium are hazardous to people and especially sensitive in young children. Even though zinc and copper are necessary heavy metals with a variety of biological functions, consuming these metals in excess might be harmful [3], [4], [5].

Since some metals can cause oils to oxidize, the amount of metals in edible oils affects their quality and shelf life [6]. Metals can enter the food chain via inhalation or consumption. Research suggests that certain plant species can acquire metals more efficiently than other species [7].

Certain types of cancer, neurological disorders, genetic abnormalities, infertility, renal failure, and cardiovascular illnesses are just a few of the major health problems that heavy metals can cause [3].

All endogenous factors related to plant metabolism and external factors resulting from contamination during agronomic production techniques, oil seed collection during oil extraction and treatment methods, packaging and storage systems, and materials are responsible for the presence of heavy metals in edible oils [8].

Inorganic lead and lead compounds are probably carcinogenic to humans (Group 2 A) and cadmium and cadmium compounds are carcìnogenic to humans (Group 1) [9], [10].

The most harmful heavy metals are lead and cadmium. As a human carcinogen, cadmium causes lung cancer, renal disease, liver problems, and cardiovascular system disorders. Lead is more likely to cause harm to the central and peripheral nerve systems, which may lower children's intellectual potential [3].

Institute of Standards and Industrial Research of Iran (ISIRI) recommends 80 µg/ kg for the maximum lead level for edible vegetable oils (11). According to the regulation (EU) 2023/915 on maximum levels for certain contaminants in food, the maximum lead level for oils is 100 µg/ kg [12]. There is no maximum residue limit (MRL) for cadmium in vegetable oils by Iranian and European standards.

A study conducted in Hamadan, Iran, assessed the presence of potentially toxic elements including lead (Pb), arsenic (As), cadmium (Cd), iron (Fe), and zinc (Zn) in traditional and industrial edible vegetable oils (peanut, sunflower, olive, and sesame). The study found that the concentrations of these elements were higher in industrially produced vegetable oils than in those produced traditionally [13]. A study conducted in Northern Cyprus investigated the content of trace metals, including copper (Cu), cadmium (Cd), chromium (Cr), arsenic (As), lead (Pb), and nickel (Ni) in different varieties of olive oils. The study found that heavy metals can have a negative effect on the oxidative rate of the oils and can be toxic to consumers. The concentrations of these metals in olive oil were found to be within the MRL [14].

A comparative investigation of heavy metal levels in edible vegetable oils was conducted. The researchers examined more than 25 heavy metals in 35 different oils from 24 nations, including Iran. The most commonly researched metals were Cd, Pb, Cu, and Fe in olive, sunflower, rapeseed, and corn oils [15]. A study conducted in Northern Iran investigated the lead and cadmium content of Korbal rice. The study found that the pollution of the river had a significant effect on the lead and cadmium content of the rice samples [16]. Research done in Iran looked at the amounts of Pb, Cd, and As in groundwater samples. The analysis found that the mean Pb and Cd levels in groundwater samples were 2.6 µg/L and 0.9 µg/L, respectively [17]. A health risk evaluation of lead, cadmium, and arsenic in leafy vegetables in Tehran, Iran was carried out. The study discovered that contaminants such as heavy metals have directly affected public health and led to new diseases in recent years [18].

In research, the levels of eight heavy metals (Cu, Zn, Fe, Mn, Cd, Ni, Pb, As) in nine types of edible vegetable oils from China were analyzed using advanced spectroscopic techniques. The accuracy of the procedure was confirmed using certified reference materials. The results showed that the concentrations of these metals varied within a certain range. Overall, the iron content was found to be higher than the other metals in the oils. Based on recommended safety intake levels, consuming these oils in specified amounts should not pose a risk to human health [19].

In Italy, Dugo et al. determined the levels of Cd(II), Pb(II), Cu(II), and Zn(II) in different commercial oils using derivative potentiometric stripping analysis. The analysis of commercial oils revealed that cadmium levels were below 4.90 µg /kg, lead levels ranged from 8.60 to 55.61 µg /kg, copper levels ranged from 53.80 to 674.45 µg /kg, and zinc levels ranged from 51.45 to 555.61 µg /kg [20].

The MRL of lead in corn oil and olive oil is 80 µg/kg according to the Iranian national standard, and no standard has been defined for the permissible limit of residual cadmium in vegetable oils [11].

This study aimed to investigate the levels of heavy metal contamination in various edible oils in Gilan province, Iran, and assess the associated health risks. This research is crucial due to the increasing concerns about food safety and the lack of comprehensive data on heavy metal contamination in this region. Our innovative approach includes a detailed risk assessment using BMDL10 and MOE methods, providing novel insights into the potential health impacts of these contaminants.

## 2 Materials and method

### 2.1 Sample collection

For this study, from March to June 2021, 30 samples of olive oil (12 samples of commercial olive oil and 18 traditional olive oil) and 30 samples of corn oil (18 samples of commercial corn oil and 12 traditional corn oil) were collected randomly from sales centers in Gonbad-Kavus city. The number of each type of sample is determined based on the number of oil supply centers in Gonbad-Kavus city. The olive oil analyzed was extra virgin. The commercial oils (3–5 Trademark) were produced in Iran. The traditional oils were produced in local stores in Gonbad-Kavus city.

The samples were sent to the biochemistry laboratory of Kharazmi University and the amounts of lead and cadmium were determined by the graphite furnace atomic absorption spectrometry method.

Gonbad-Kavus city is in Golestan province, the northern part of Iran. At the 2016 census, the population was 348744.

### 2.2 Reagents and microwave digestion

The nitric acid utilized for sample preparation was of ultrahigh purity. The laboratory gear, including pipette tips and autosampler cups, was thoroughly cleaned with detergent and tap water, rinsed with distilled water, immersed in dilute nitric acid, and rinsed with deionized distilled water. All reagents and standard stock solutions were prepared from Merck (Germany) [19], [21].

Standard solutions were made by diluting stock standard solutions (1000 mg/L) in a 0.1 % v/v nitric acid solution just before use [19], [21].

Samples weighing 1 g were digested in a microwave with 6 ml of concentrated HNO_3_ (65 %) and 2 ml of concentrated H_2_O_2_ (30 %) for 32 minutes before being diluted to 10 ml with 2 % HNO_3_. Each sample solution was clear. A blank digest was performed in the same method. The microwave system's digestion conditions were 3 minutes for 500 W, 5 minutes for 800 W, 8 minutes for 1000 W, and 10 minutes for 1300 W, with an 8-minute vent time [19], [21].

### 2.3 Determination of lead and cadmium

For the analysis, a Varian SpectrAA-200 system (Australia) with deuterium background correction was used. A programmable sample dispenser and a Varian GTA-110 partition graphite tube atomizer were included with the spectrometer. High-purity argon was the inert gas. The wavelength for lead and cadmium were 228.8 and 283.3 nm respectively. The lamp intensity for lead and cadmium were 3 and 4 mA respectively. To detect lead and cadmium in vegetable oils, the chemical modifier NH_4_H_2_PO4/Mg(NO_3_)_2_ was utilized.

The mode of measurement used was peak height. Aqueous working lead and cadmium standards (10, 20, 30, 40, and 60 μg/L) prepared from Pb and Cd stock standard solution (1000 mg/L) were used to calibrate the instrument. Three replicate readings were obtained for standard solutions, and the injection volume remained constant at 10 μL.

### 2.4 Noncancer risk assessment

#### 2.4.1 Estimated daily intake (EDI) of metals

The EDI of metals was evaluated by considering the daily consumption of 0.147 and 0.328 g for corn and olive oils in Iran respectively [22], [23]. The body weight of adults was set to 70 kg. The EDI was calculated according to the following equation [11], [24].

$$\mathrm{EDI}=\frac{\mathrm{Daily}\;\mathrm{oil}\;\mathrm{consumption}\;(\mathrm{kg})\times \mathrm{Mean}\;\mathrm{concentration}\;\mathrm{of}\;\mathrm{metal}\;(\mathrm{mg}/\mathrm{kg})}{\mathrm{Average}\;\mathrm{body}\;\mathrm{weight}\;(\mathrm{kg})}$$

#### 2.4.2 Hazard Quotient (HQ)

The HQ for the non-carcinogenic risk of heavy metal can be calculated as the following equation:

$$\mathrm{HQ}=\frac{\mathrm{EDI}}{\mathrm{RfD}}$$

According to the U.S. Environmental Protection Agency (EPA), the oral toxicity reference dose value (RfD) is 1 µg/kg for cadmium (EPA 2016). The RfD for lead in this study was 3.75 µg/kg calculated from the published paper in 2021 [24].

#### 2.4.3 Hazard Index (HI)

Exposure to two or more heavy metals may enhance the interaction effects. The HI, which represents the entire non-carcinogenic health risk of heavy metals, was calculated as follows: [24]

HI = HQ_1_ + HQ_2_ + HQ_3_ + … + HQ_n_

### 2.5 Cancer risk assessment

According to EFSA recommendations, margins of exposure (MOE) were calculated. The BMDL10 (Benchmark Dose with 10 % effect) was used in an animal cancer experiment. The BMDL10 values for lead and cadmium were 0.63 and 0.36 µg/kg body weight/day, respectively, which have been established based on reduced kidney function in adults [25]. The EDI was calculated according to the following equation:

$$\mathrm{MOE}=\frac{\mathrm{BMDL}10}{\mathrm{EDI}}$$

A lower MOE (e.g., below 100) indicates a higher risk. Human exposure is closer to the dose that causes adverse effects in animals [26].

### 2.6 Statistical analyses

The levels of lead and cadmium in vegetable oil samples were compared using SPSS software (version 24) and GraphPad Prism 10.2.1 (GraphPad Software, Boston, Massachusetts, USA) with ANOVA. Duncan’s multiple comparison test was utilized. A two-tailed *P* value of 0.05 or less indicated statistical significance.

## 3 Results

**Fig. 1:**
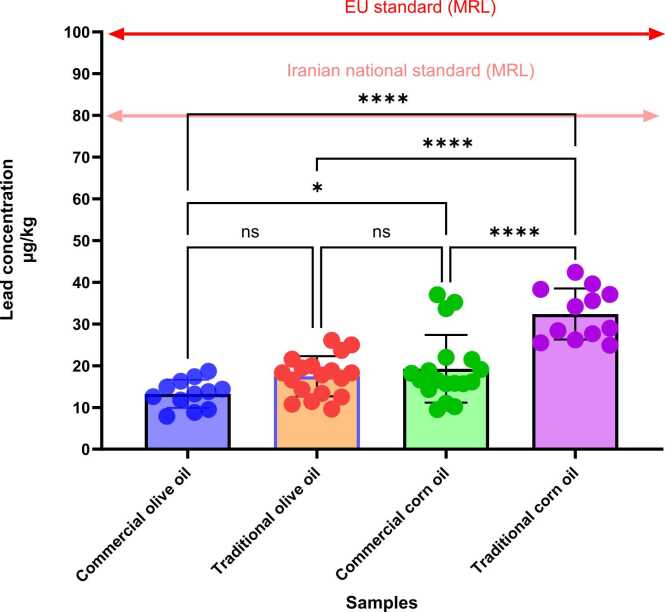
Levels of lead concentration in olive and corn oil samples (n = 60) collected from Gonbad-Kavus city, Iran (the MRL for lead in vegetable oils is 80 µg/kg, as established by the Iranian national standard)(ISIRI, 2021). Significance in lead concentration among different oil types is labeled by ^ns^: P value > 0.05 * : P value < 0.05 * *: P value < 0.01 * ** : P value < 0.001 or * ** *: P value < 0.0001 according to the one-way ANOVA test.

**Fig. 2:**
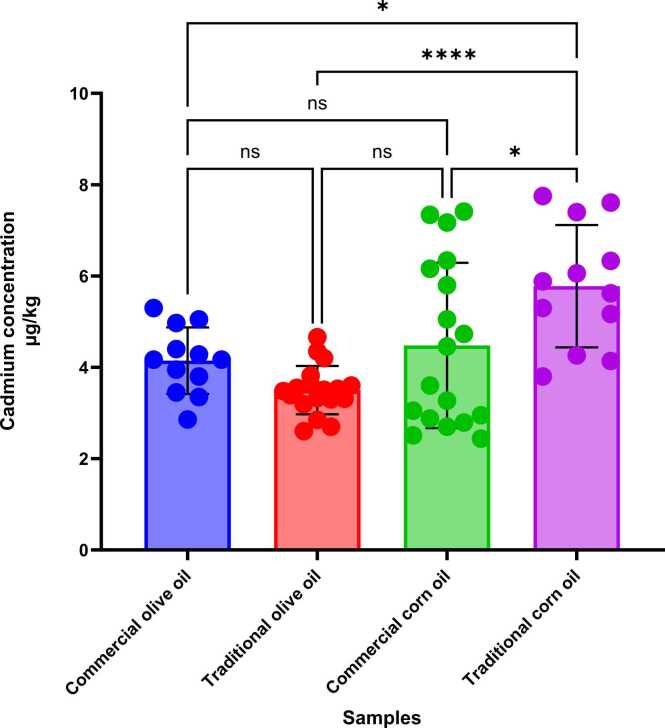
Levels of cadmium concentration in olive and corn oil samples (n = 60) collected from Gonbad-Kavus city, Iran. Significance in cadmium concentration among different oil types is labeled by ^ns^: P value > 0.05 * : P value < 0.05 * *: P value < 0.01 * ** : P value < 0.001 or * ** *: P value < 0.0001 according to the one-way ANOVA test.

Lead and cadmium residue was seen in all samples. The results obtained from the analysis of vegetable oils showed that the concentrations of lead in commercial olive oil, traditional olive oil, commercial corn oil, and traditional corn oil were 13.27 ± 3.37, 17.48 ± 4.82, 19.27 ± 8.12, and 32.40 ± 6.13 μg/kg, respectively. The concentrations of cadmium in commercial olive oil, traditional olive oil, commercial corn oil, and traditional corn oil were 4.14 ± 0.53, 3.50 ± 0.72, 4.48 ± 1.80, and 5.77 ± 1.34 µg/kg, respectively (Table 1)(Fig. 1, Fig. 2).

**Table 1:** Comparison and ranges of the means of lead and cadmium concentrations in various oils samples collected in Gonbad-Kavus city, Iran.

	Lead µg/kg			Cadmium µg/kg		
Products	Mean ± SD	Min	Max	Mean ± SD	Min	Max
Traditional corn oil	32.40 ± 6.13^a^	24.90	42. 40	5.77 ± 1.34^a^	3.80	7.75
Commercial corn oil	19.27 ± 8.12^b^	9.50	37	4.48 ± 1.80^b^	2.44	7.40
Traditional olive oil	17.48 ± 4.82^bc^	9.60	26.10	3.50 ± 0.72^c^	2.60	4.66
Commercial olive oil	13.27 ± 3.37^c^	7.90	18.70	4.14 ± 0.53^bc^	2.86	5.30
SEM	1.13			0.18		
*P* value	0.0001			0.0001		

Means with the same code are not significantly different.

SD: Standard Deviation

SEM: Standard Error of the Mean

According to Table 1, the highest lead and cadmium concentrations were in the traditional corn oil. Institute of Standards and Industrial Research of Iran (ISIRI) recommends 80 µg/ kg for the maximum lead level for edible vegetable oils [11]. The lead level in all investigated edible oils was found to be lower than MRL (Fig. 1).

Acceptable daily intake for lead and cadmium according to the EFSA are 3 and 1 µg/kg bw/day (26).

Table 2 shows the Estimated Daily Intakes (EDI), Hazard Quotient (HQ), Hazard Index (HI), and MOE of lead (Pb) and cadmium(Cd) in olive and corn oils.

**Table 2:** Estimated Daily Intakes (EDI), Hazard Quotient (HQ), Hazard Index (HI), and margins of exposure (MOE) of lead (Pb) and cadmium(Cd) in olive and corn oils from consumption of 40.2 g/day in Gonbad-Kavus city, Iran.

Risk assessment		Commercial corn oil	Traditional corn oil	Commercial olive oil	Traditional olive oil
EDI	Pb	0.00000004	0.00000006	0.00000006	0.00000008
	Cd	0.000000009	0.00000001	0.00000001	0.00000001
HQ	Pb	0.000010	0.000016	0.000016	0.000021
	Cd	0.000009	0.00001	0.00001	0.00001
HI^a^	∑HQ	0.000019	0.000026	0.000026	0.000031
MOE^b^	Pb	15750	10500	10500	7875
	Cd	40000	36000	36000	36000

a HI< 1 indicates a low level of non-cancer risk.

b MOE< 10000 indicates a concern for potential cancer risk.

The HI levels for lead and cadmium residues in various oils were below 1, indicating no non-carcinogenic risk.

A MOE of 10,000 or higher is generally considered to indicate low concern for carcinogenic substances. This means that the level of exposure is deemed to be low enough not to pose a significant risk to human health. Based on the results of the study, the MOE value in traditional olive oil for the lead was less than 10,000, whereas in other oils, it was more than 10,000, indicating no significant risk to consumers.

## 4 Discussion

The minimum and maximum lead levels were 7.90 µg/kg in commercial olive oil and 42.40 µg/kg in traditional corn oil. The result of the study showed that the lead levels in Gonbad-Kavus were higher than the results in previous studies in Iran [21].

Pehlivan et al.(2008) in Turkey, showed that the highest lead concentration was in virgin olive oil and cadmium was in sunflower oil [27]. Lead causes health issues such as insomnia, fatigue, and weight loss [19]. Cadmium, a highly hazardous element found naturally in soil, is also released into the environment through human activities. Excessive cadmium exposure can cause renal, pulmonary, hepatic, skeletal, and reproductive problems, as well as cancer [19]. In Croatia, the vegetable oils were analyzed, and the lead and cadmium concentrations were lower than 1 µg/kg which was in contrast to current results [28].

In Turkey, Acar (2012) showed that the lead and cadmium concentrations in olive oil were 0.25 and 51 µg/kg, respectively [29].

A research result in Pakistan showed that the lead and cadmium levels in the sunflower oil were in the range of 0.79–4.29 and 1.70–6.18 µg/kg, respectively [30] which the lead level was lower than the current study and the cadmium level was similar to the current result.

Dugo et al.(2004) determined the content of Cd, Pb, Cu, and Zn in commercial peanuts, sunflower, soy, maize, rice, grape seed, and hazelnut oils in Italy. The lead and cadmium levels were in the range of 5.7–10.9 and 2.58–6.72 µg/kg, respectively [20]. The lead level in the current study was higher than the results of Dugo et al. Particularly the obtained results provide evidence that corn oil is a good source of lead in the north of Iran.

A vital area of food safety research is the hazard index and risk assessment of heavy metals in edible oils. Edible oils may contain heavy metals from a variety of sources, such as the soil, fertilizers, manufacturing processes, and even packaging materials [15].

Embaby et al., studied toxic metal levels (Hg, Pb, Cd, and As) in 96 samples of sardines and shrimp from four Egyptian coastal governorates (Alexandria, Kafr El-Sheikh, Damietta, and Port Said) from 2019 to 2021, using inductively coupled plasma-optical emission spectroscopy (ICPOES). Mercury was not detected in any samples. Lead and cadmium levels were higher in winter than in summer, while arsenic levels remained constant across seasons. The health risk from these metals was low, except for sardine samples from Kafr El-Sheikh, which showed a higher risk [31].

Li and Zhang conducted a study on the intake of lead and cadmium by Belgian consumers from ceramic ware. They utilized detailed scenarios to examine metal release and usage. Initially, they made a direct estimation of metal intake, followed by a probabilistic estimation if the direct estimation exceeded toxic limits. The risk was assessed through the MOE and tolerable weekly intake (TWI). For adults, the median and 95th percentile lead intakes were 0.02 and 5.77 μg/kg body weight per day, respectively, while for children, they were 0.07 and 17.3 μg/kg body weight per day. Cadmium intake followed a similar pattern. The study revealed that exposure levels could exceed the TWI by up to 20 times, highlighting the necessity to reduce migration limits for ceramic ware to mitigate health risks [32].

Taghizadeh et al., studied 1800 Iranian olive samples from 20 cultivars across six zones, measuring eight metals. They found no significant differences among samples. Health risks from consumption were low, with hazard quotients below 1 [33]. Our findings align with this study and the HI was lower than 1.

Taghizadeh et al., assessed risks from heavy metals in 375 vegetable oil samples using ICP-OES methods. Incremental lifetime cancer risk (ILCR) analysis showed potential risk for arsenic among heavy metals but not for non-carcinogenic risks. Sensitivity analyses indicated that analyte concentration most influenced risk assessments [26].

Our findings align with previous studies, such as Taghizadeh et al. and Embaby et al., which reported similar risk assessments of heavy metal contamination in food products.

Raeeszadeh et al. examined heavy metal levels in the meat of sheep, beef, turkeys, and ostriches in Sanandaj, Kurdistan, Iran. Researchers tested 170 meat samples for metals like selenium, lead, cadmium, arsenic, cobalt, zinc, nickel, copper, and chromium using the ICP-MS method. Results indicated no significant differences in selenium, nickel, cobalt, and chromium levels, but lead, cadmium, arsenic, zinc, copper, chromium, and nickel exceeded MRL [2]. In the current study, the level of lead in oil samples was lower than MRL.

A metric used to evaluate the possible risk associated with prolonged exposure to several heavy metals is the Hazard Index. The Hazard Quotient, which is the ratio of a metal's estimated daily intake to its reference dose, is added up for each metal. It is generally accepted that there is no appreciable risk if the HI is less than 1. On the other hand, a potential health risk is indicated if the HI is greater than 1. The dietary intake of heavy metals from daily consumption of edible vegetable oils for a 70 kg individual should pose no risk to human health in Gonbad-Kavus city [34].

Based on the results of the study, the MOE value in traditional olive oil for the lead was less than 10,000, whereas in other oils, it was more than 10,000, indicating no significant risk to consumers. It is recommended to review national standards or increase monitoring of lead and cadmium sources in vegetable oils.

## 5 Conclusion

The analysis of lead and cadmium concentrations in commercial and traditional olive and corn oils from Gonbad-Kavus City revealed detectable levels of both heavy metals. All measured concentrations were below the MRL established by the Institute of Standards and Industrial Research of Iran (ISIRI). Hazard Quotient (HQ) and Hazard Index (HI) values indicated no non-carcinogenic health risks associated with the consumption of these oils. However, the Margin of Exposure (MOE) for lead in traditional olive oil was found to be less than 10,000, highlighting a potential risk that warrants further investigation. This study was limited by the sample size and geographic scope, focusing solely on Gonbad-Kavus City. Future research should encompass a broader range of samples and locations to provide a more comprehensive assessment of heavy metal contamination in vegetable oils. Promoting public awareness through campaigns and providing clear guidelines play a critical role in minimizing heavy metal exposure. Additionally, maintaining regular monitoring, stringent quality control, and strict adherence to safety standards are essential practices within the oil industry in Iran.

## CRediT authorship contribution statement

**Movassaghghazani Mohammadhosein:** Writing – review & editing, Writing – original draft, Supervision, Project administration, Methodology, Conceptualization. **Tayeb Janan:** Investigation, Data curation.

## Declaration of Competing Interest

The authors declare that they have no known competing financial interests or personal relationships that could have appeared to influence the work reported in this paper.

## Data Availability

The authors do not have permission to share data.
